# Chondrocyte Behavior on Micropatterns Fabricated Using Layer-by-Layer Lift-Off: Morphological Analysis

**DOI:** 10.1155/2013/560328

**Published:** 2013-05-28

**Authors:** Jameel Shaik, Javeed Shaikh Mohammed, Michael J. McShane, David K. Mills

**Affiliations:** ^1^Institute for Micromanufacturing, Louisiana Tech University, Ruston, LA 71272, USA; ^2^Biomedical Engineering Program, Louisiana Tech University, Ruston, LA 71272, USA; ^3^School of Bio Sciences & Technology, VIT University, Vellore 632014, India; ^4^Biomedical Technology Department, King Saud University, Riyadh 11433, Saudi Arabia; ^5^Biomedical Engineering Program, Texas A&M University, College Station, TX 77843, USA; ^6^School of Biological Sciences, Louisiana Tech University, Ruston, LA 71272, USA

## Abstract

Cell patterning has emerged as an elegant tool in developing cellular arrays, bioreactors, biosensors, and lab-on-chip devices and for use in engineering neotissue for repair or regeneration. In this study, micropatterned surfaces were created using the layer-by-layer lift-off (LbL-LO) method for analyzing canine chondrocytes response to patterned substrates. Five materials were chosen based on our previous studies. These included: poly(dimethyldiallylammonium chloride) (PDDA), poly(ethyleneimine) (PEI), poly(styrene sulfonate) (PSS), collagen, and chondroitin sulfate (CS). The substrates were patterned with these five different materials, in five and ten bilayers, resulting in the following multilayer nanofilm architectures: (PSS/PDDA)_5_, (PSS/PDDA)_10_; (CS/PEI)_4_/CS, (CS/PEI)_9_/CS; (PSS/PEI)_5_, (PSS/PEI)_10_; (PSS/Collagen)_5_, (PSS/Collagen)_10_; (PSS/PEI)_4_/PSS, (PSS/PEI)_9_/PSS. Cell characterization studies were used to assess the viability, longevity, and cellular response to the configured patterned multilayer architectures. The cumulative cell characterization data suggests that cell viability, longevity, and functionality were enhanced on micropatterned PEI, PSS, collagen, and CS multilayer nanofilms suggesting their possible use in biomedical applications.

## 1. Introduction

Replicating the highly structured *in vivo* microenvironment is crucial in understanding cellular behavior [1]. Traditional cell culture surfaces cannot provide sufficient control over the cellular microenvironment [2] for use in studying many anchorage-dependent cellular processes such as cellular differentiation, proliferation, and phenotypic expression. Cell supportive substrates, with the requisite spatiotemporal surface properties, are also a critical feature in designing appropriate biomaterial surfaces for use in cell arrays, bioreactors, biosensors [3], and cocultures [4–6] and for use in engineering new tissues for repair or replacement.

Micropatterned surfaces have been explored as a means not only to answer fundamental questions in cell biology but also to develop cell culture substrates with surface features tailored for specific bio- and tissue engineering applications [2, 3, 7]. This was demonstrated by the growth of hepatocytes on micropatterned surfaces [4, 5]. The authors observed decreased DNA production and increased cellular apoptosis associated with a decrease in the adhesiveness of the surfaces [7]. Cell shape was also found to be the regulatory factor in both cell apoptosis and growth [7, 8]. This was achieved by an increasing restriction of the size of micropatterned islands coated with different densities of ECM and growing bovine and human endothelial cells on these islands [2].

Patterning cells using cell-adhesive [9–13] or cell-repulsive [14–20] surfaces or combinations [21, 22] of adhesive and nonadhesive surfaces have been developed, and a wide variety of eukaryotic cells have been grown and studied on these micropatterned surfaces [2, 5, 8, 20, 23–25]. A broad range of materials have been used in creating these micropatterned cell culture surfaces [3, 8, 26, 27]. 

Micropatterned substrates have also lent credence to the important understanding that the degree of cellular contraction is crucial in determining a cells fate during differentiation, especially in the case of stem cells [1]. This has been demonstrated in several studies that showed that variation in micropattern size directed stem cell differentiation into different cell lineages. For example, human mesenchymal stem cells (hMSCs) cultured in differentiating medium exhibited differences in the contraction levels and also exhibited different lineages—those hMSCs grown on 1,000 *μ*m^2^ micropatterns had low contraction levels and differentiated into adipocytes, while hMSCs plated on 10,000 *μ*m^2^ micropatterns were highly contracted and differentiated into osteoblasts [28]. Similarly, hMSCs treated with transforming growth factor *β* (TGF-*β*) exhibited differential behavior dependent upon the size of micropatterns—hMSCs plated on small micropatterns differentiated into chondrocytes, while hMSCs plated on large micropatterns differentiated into myocytes [29]. 

The use of layer by layer (LbL) nanoassembly for creating micropatterned surfaces brings in all the advantages offered by LbL—simplicity and excellent control over surface properties such as thickness, roughness, and porosity [3]. LbL surfaces can potentially be used in obtaining the precise cellular microenvironment as the surfaces can be tuned to release the factors necessary for the growth and regulation of cells [22]. Polyelectrolytes and proteins deposited through the LbL technique can be used to create either cell-resistant or cell-adhesive micropatterns. Our previous studies focused on the growth and behavior of bovine articular chondrocytes [30], human chondrosarcoma cells, and canine chondrocytes [31] on LbL-assembled nanothin films of varying configurations. We chose chondrocytes as our model cell type as they have a very plastic phenotype. Cell characterization studies were used to assess chondrocyte viability, longevity, and functionality in response to the configured architectures. Cell adhesion, shape, and functionality are linked to the nature of the underlying culture substrate [32, 33]. 

Our goal in this study was to expand our previous work by examining interspecies differences in chondrocyte behavior on micropatterned substrates created using the LbL-LO method. Our expectation was that difference in nanofilm architectures atop micropatterned substrates would evoke variations in chondrocyte behavior. Different micropatterned surfaces were created using the LbL-LO technique [6, 34–36]. Based on our previous studies, five polyelectrolytes/proteins were used to construct the nanofilms [31]. These were poly(dimethyldiallylammonium chloride) (PDDA), poly(ethyleneimine) (PEI), poly(styrene sulfonate) (PSS), collagen, and chondroitin sulfate (CS). The substrates were patterned, in five and ten bilayers, resulting in the following multilayer nanofilm architectures: (PSS/PDDA)_5_, (PSS/PDDA)_10_; (CS/PEI)_4_/CS, (CS/PEI)_9_/CS; (PSS/PEI)_5_, (PSS/PEI)_10_; (PSS/Collagen)_5_, (PSS/Collagen)_10_; (PSS/PEI)_4_/PSS, (PSS/PEI)_9_/PSS. 

## 2. Materials and Methods

### 2.1. Substrates

Microscope cover slips (Thickness number 2, 18 × 18 mm^2^, Electron Microscopy Sciences, Hatfield, PA, USA) were used as the substrates for deposition of the micropatterns. These substrates were chosen for ease in optical characterization. 

### 2.2. Chemicals

Nano-Strip from CYANTEK Corporation (Fremont, CA); positive photoresist S1813 and positive resist developer MF-319 from the Shipley Corporation (Marlboro, Massachusetts) were used. All the chemicals were purchased from Sigma-Aldrich unless otherwise specified. All commercial chemicals were used following manufacturer's directions. 

### 2.3. Preparation of Polyelectrolyte, Polypeptide, and Protein Solutions

PDDA (Mw ~ 150 kDa), PSS (Mw ~ 1 MDa) solutions were prepared at concentrations of 2 mg mL^−1^ with 0.5 M KCl, and a PEI (Mw ~ 750 kDa) solution of 2 mg mL^−1^ was prepared in deionized (DI) H_2_O for use in LbL nanoassembly. Chondroitin sulfate (Mw ~ 500 Da) and type I collagen (Mw ~ 100 kDa) (Cohesion, Palo Alto, CA, USA) were prepared at a concentration of 120 *μ*g mL^−1^. All solutions were prepared using DI water with a resistivity of 18.2 MΩ cm (Millipore systems, Burlington, MA, USA). 

### 2.4. Substrate Pretreatment

The substrates were first incubated in Nano-Strip at 70°C for 1 h followed by rinsing in DI water anddried in a N_2_ stream to remove any organic materials and to create a uniform negative charge on the substrates. A precursor layer of PDDA was then deposited onto the substrates to render a cytophobic background on the substrates. This was based on our previous results with smooth muscle and neuronal cells [35, 37]. PDDA application is not exclusive as any cytophobic material other than PDDA can also be used.

### 2.5. Photolithography

To help withstand the centrifugal forces during spin coating, the PDDA-coated glass substrates were attached to silicon wafer pieces using photoresist S1813 and heated at 165°C for 5 min to hard bake the photoresist. Next, positive photoresist S1813 was spun (1000 rpm-100 r s^−1^-10 s, 3000 rpm-500 r s^−1^-50 s) on the PDDA-coated substrates, soft baked at 115°C for 1 min, and photo-patterned using UV radiation (400 nm, 7 mW cm^−2^) applied for 18 s. The mask used for pattern transfer contained 80 *μ*m wide stripe patterns separated by 240 *μ*m and 100 *μ*m wide stripe patterns separated by 300 *μ*m. Finally, the patterns were developed for 15 s using MF-319, and the substrates quickly rinsed in DI water and dried using N_2_. 

### 2.6. Layer-by-Layer (LbL) Self-Assembly

Micropatterned substrates were then modified using LbL nanoassembly. The substrates were dipped in polyelectrolyte and protein solutions for 10 min and 30 min, respectively. PSS or PEI was used as the polyanions in all multilayer nanofilm configurations. After every deposition step, substrates were rinsed in DI water and then dried using N_2_. Substrates were patterned with PDDA, PSS, PEI, collagen, and CS as either five- or ten-bilayer nanofilms. Thus, the following configurations were fabricated: (PSS/PDDA)_5_, (PSS/PDDA)_10_; (CS/PEI)_4_/CS, (CS/PEI)_9_/CS; (PSS/PEI)_5_, (PSS/PEI)_10_; (PSS/Collagen)_5_, (PSS/Collagen)_10_; (PSS/PEI)_4_/PSS, (PSS/PEI)_9_/PSS.

### 2.7. Lift-Off

Lift-off was performed by sonicating the substrates in acetone for 5 to 10 minutes. The photoresist and the nanofilms deposited on the photoresist were removed during the lift-off process. Also, the cover slip glass was detached from the silicon wafer. Surprisingly, the use of acetone was shown not to affect the biological functions of the molecules used in the LbL-LO process [38].

### 2.8. Cell Culture

Canine chondrocytes (CnC) were obtained from Cell Applications, Inc. (San Diego, CA, USA). Chondrocytes were isolated from normal canine articular cartilage and obtained at second passage. Their phenotype is preserved through ten population doublings. Chondrocytes were grown as monolayers and maintained in Chondrocyte Growth Medium (Cell Applications, Inc., San Diego, CA, USA) until the necessary cell numbers were obtained. Canine chondrocytes from passage three were used for the cell characterization studies on the micropatterned surfaces.

### 2.9. Cell Characterization

Phase-contrast microscopy was used to demonstrate the successful creation of the micropatterns. Phase-contrast microscopy was also used for the characterization of CnC on the micropatterned surfaces.

## 3. Results

### 3.1. Phase-Contrast Microscopy of Micropatterned Substrates

**Figure 1:**
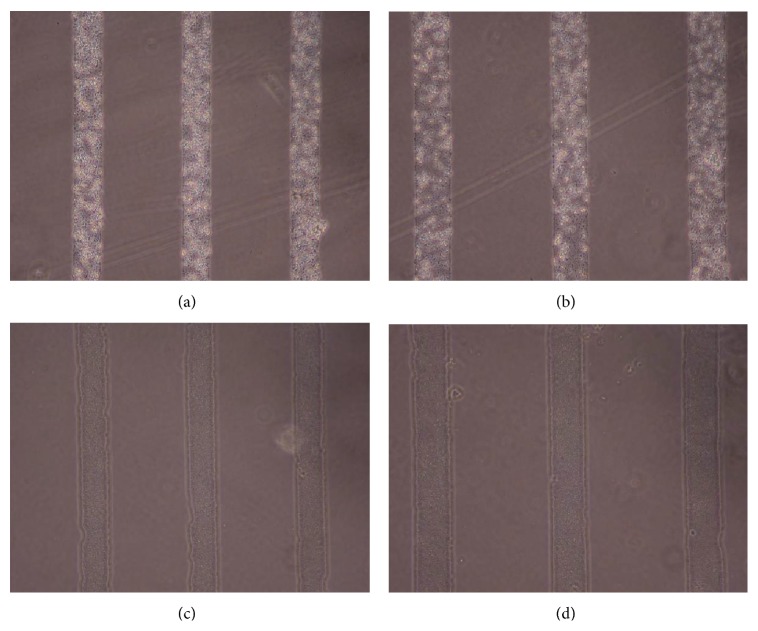
Micropatterned substrates with PDDA as the outermost layer: (a) (PSS/PDDA)_5_—80 *μ*m, (b) (PSS/PDDA)_5_—100 *μ*m, (c) (PSS/PDDA)_10_—80 *μ*m, and (d) (PSS/PDDA)_10_—100 *μ*m.

**Figure 2:**
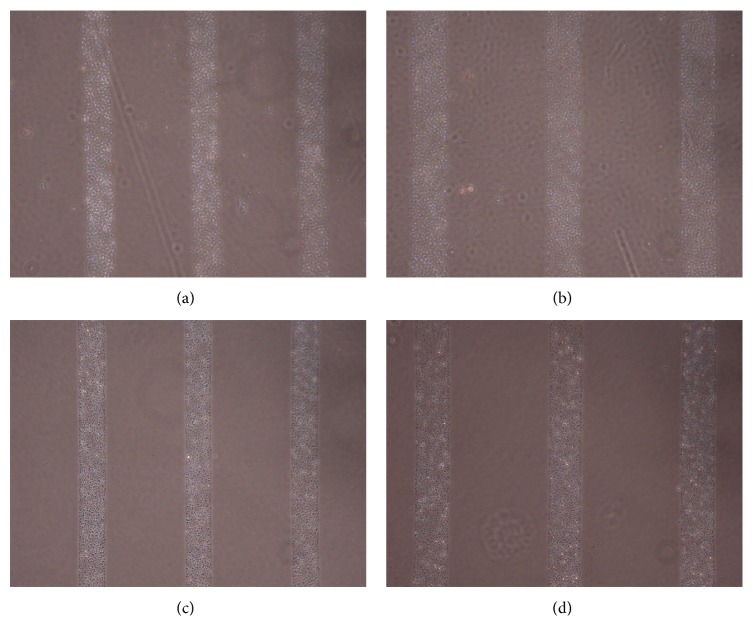
Micropatterned substrates with CS as the outermost layer: (a) (CS/PEI)_4_/CS—80 *μ*m, (b) (CS/PEI)_4_/CS—100 *μ*m, (c) (CS/PEI)_9_/CS—80 *μ*m, and (d) (CS/PEI)_9_/CS—100 *μ*m.

**Figure 3:**
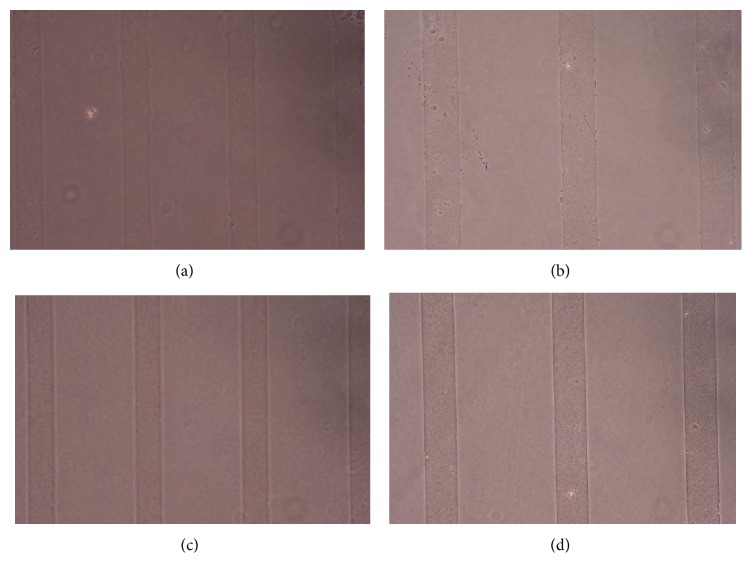
Micropatterned substrates with PEI as the outermost layer: (a) (PSS/PEI)_5_—80 *μ*m, (b) (PSS/PEI)_5_—100 *μ*m, (c) (PSS/PEI)_10_—80 *μ*m, and (d) (PSS/PEI)_10_—100 *μ*m.

**Figure 4:**
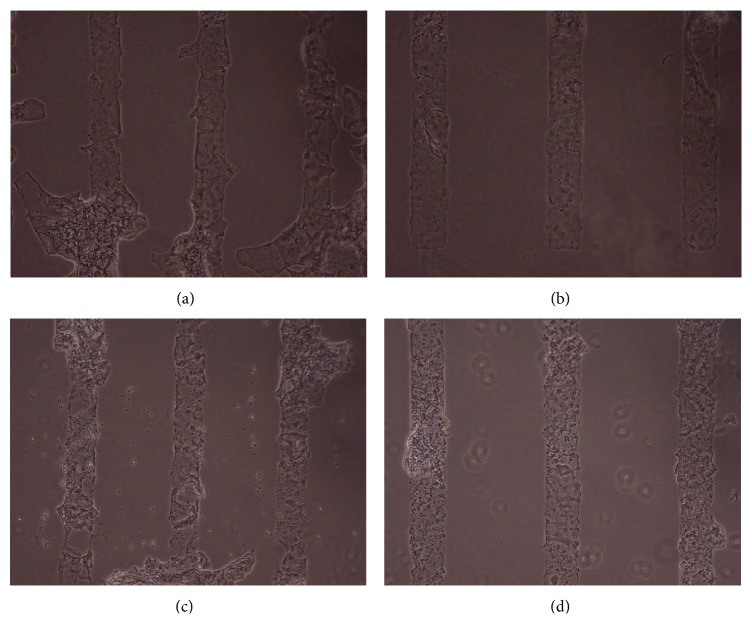
Micropatterned substrates with collagen as the outermost layer: (a) (PSS/Collagen)_5_—80 *μ*m, (b) (PSS/Collagen)_5_—100 *μ*m, (c) (PSS/Collagen)_10_—80 *μ*m, and (d) (PSS/Collagen)_10_—100 *μ*m.

**Figure 5:**
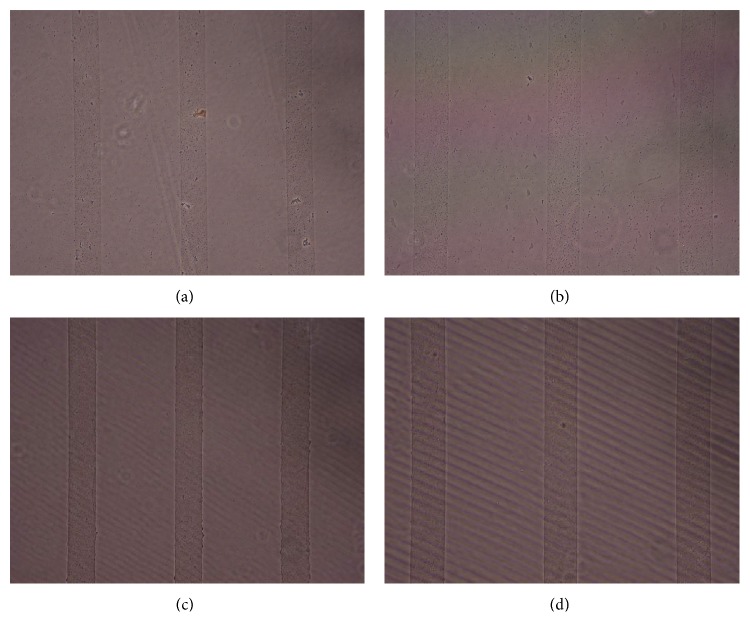
Micropatterned substrates with PSS as the outermost layer: (a) (PSS/PEI)_4_/PSS—80 *μ*m, (b) (PSS/PEI)_4_/PSS—100 *μ*m, (c) (PSS/PEI)_9_/PSS—80 *μ*m, and (d) (PSS/PEI)_9_/PSS—100 *μ*m.

Figures 1, 2, 3, 4, and 5 contain phase-contrast images of the micropatterned substrates of five different materials, in five and ten-bilayer nanofilm configurations. In all the multilayer nanofilm architectures, the terminating nanofilm layer was one of the five different materials studied here. The images show 80 *μ*m wide stripe patterns separated by 240 *μ*m or 100 *μ*m wide stripe patterns separated by 300 *μ*m.

All the micropatterns, with the exception of collagen, had high edge resolution. There could be several factors contributing to the low resolution of edges in collagen micropatterns. Some of the factors affecting the edge resolution of the collagen micropatterns could be deposition time of collagen, pH of collagen solution, sonication time during lift-off, and the height of photoresist used to define the stripe micropatterns in LbL-LO. From Figure 4, it also appears that the 100 *μ*m collagen micropatterns have better edge resolution compared to the 80 *μ*m micropatterns. One of the reasons for this difference in edge resolution of collagen micropatterns could be due to the differences in the spacing between the stripe micropatterns, which is 240 *μ*m for the 80 *μ*m stripe patterns and 300 *μ*m for the 100 *μ*m stripe patterns. From Figures 1–5, it is also clear that the 10-bilayer micropatterns appear to have better surface coverage of materials compared to their 5-bilayer counterparts.

### 3.2. Phase-Contrast Microscopy of CnC on Micropatterned Substrates

Figures 6, 7, 8, 9, 10, 11, 12, 13, 14, 15, 16, 17, and 18 contain representative phase-contrast images of the chondrocytes on micropatterned substrates of five different materials, in five- and ten-bilayer nanofilm configurations. In all the multilayer nanofilm architectures, the terminating nanofilm layer was one of the five different materials studied here, and the background material is a single layer of PDDA. 

**Figure 6:**
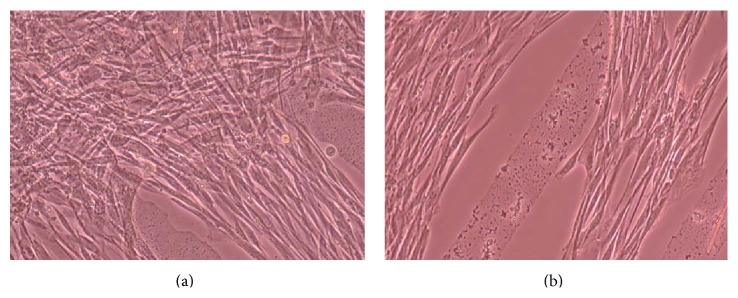
CnC on PDDA micropatterns at 3 days after seeding: (a) (PSS/PDDA)_5_—100 *μ*m, (b) (PSS/PDDA)_10_—100 *μ*m.

Figure 6 shows CnC on PDDA-terminating substrates 3 days after cell seeding. The cells appear to be avoiding the PDDA stripe patterns and are showing defined growth on the surfaces between the stripe patterns coated with a single nanofilm layer of PDDA. PDDA was shown to be a cytophobic material that inhibited smooth muscle cells and neuronal attachment [35, 37]. From our AFM measurements [39], it was observed that the thicknesses of 5 and 10 bilayers of PDDA are roughly 59.61 ± 4.07 and 251.58 ± 6.28 nm (mean ± standard deviation), respectively. Both these thicknesses are considerably greater than the thickness of a PDDA monolayer. Chondrocytes seem to prefer a thinner PDDA layer for their attachment as compared to the thicker-layered micropatterned PDDA. 

**Figure 7:**
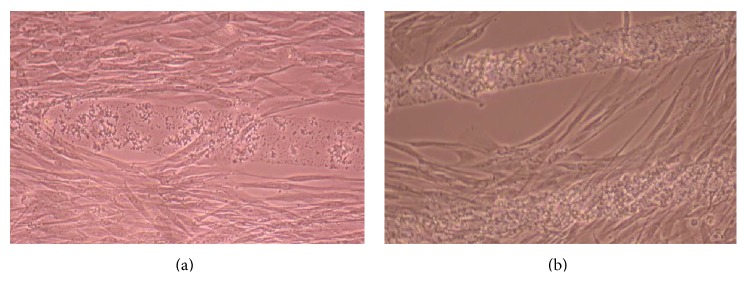
CnC on PDDA micropatterns at 10 days after seeding: (a) (PSS/PDDA)_5_—100 *μ*m, (b) (PSS/PDDA)_10_—80 *μ*m.

**Figure 8:**
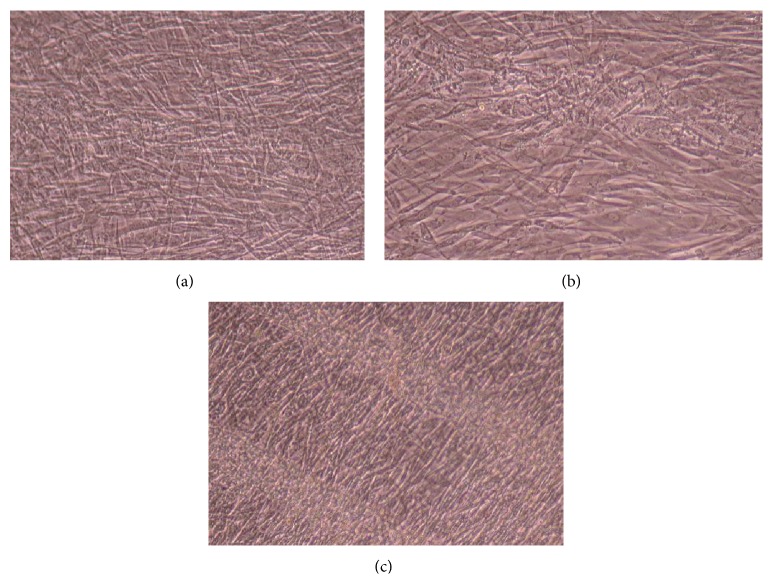
CnC on PDDA micropatterns at 11 days after seeding: (a) (PSS/PDDA)_5_—80 *μ*m, (b) (PSS/PDDA)_5_—100 *μ*m, and (c) (PSS/PDDA)_10_—80 *μ*m.

**Figure 9:**
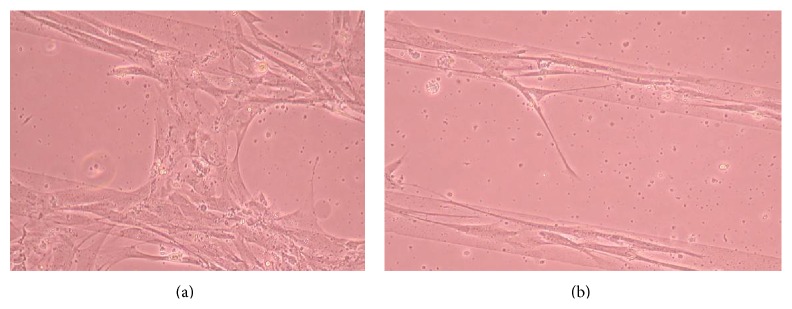
CnC on PEI micropatterns at 9 days after seeding: (a) (PSS/PEI)_10_—100 *μ*m, (b) (PSS/PEI)_10_—100 *μ*m.

**Figure 10:**
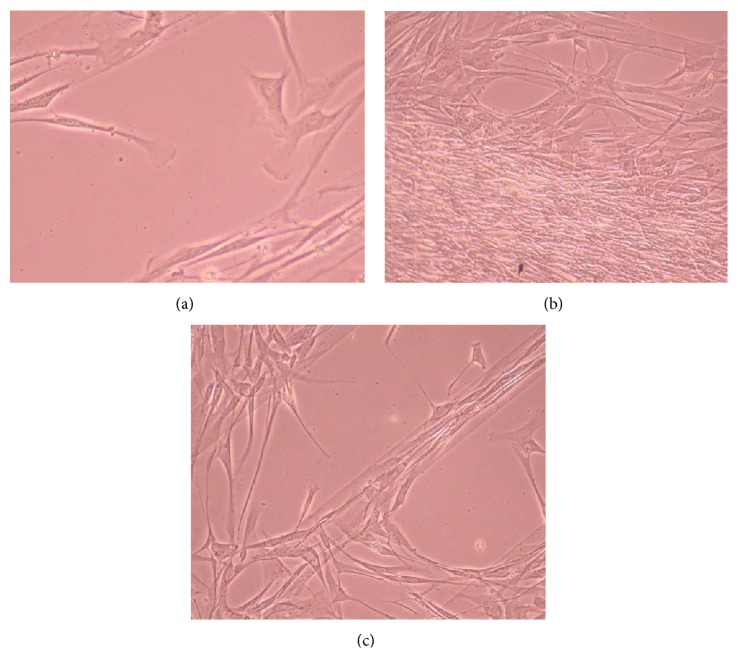
CnC on PEI micropatterns at 11 days after seeding: (a) (PSS/PEI)_5_—100 *μ*m, (b) (PSS/PEI)_10_—80 *μ*m, and (c) (PSS/PEI)_10_—100 *μ*m.

From Figure 7 it can be seen that some CnC are growing on the PDDA micropatterns. However, the growth of CnC between the micropatterns is still greater than that on the micropatterns, and it is clear that the cells have reached confluence on the PDDA micropatterns (as seen in Figure 8). From Figures 9 and 10, it can be observed that CnC are preferentially growing on the PEI micropatterns. From Figure 10(b), it is clearly shown that CnC growing on the micropatterns were aligned along the length of the stripe patterns, whereas CnC growing on unpatterned substrate are attached and growing on the monolayer of PDDA.

**Figure 11:**
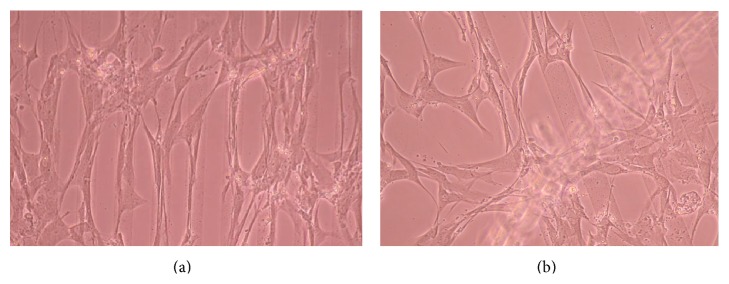
CnC on PSS micropatterns at 9 days after seeding: (a) (PSS/PEI)_4_/PSS—80 *μ*m, (b) (PSS/PEI)_4_/PSS—80 *μ*m.

**Figure 12:**
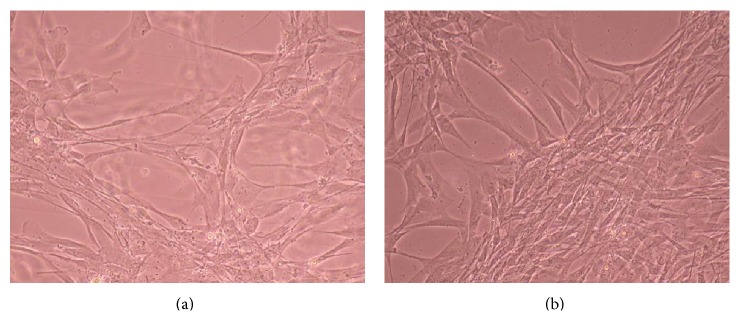
CnC on PSS micropatterns at 10 days after seeding: (a) (PSS/PEI)_4_/PSS—80 *μ*m, (b) (PSS/PEI)_4_/PSS—100 *μ*m.

**Figure 13:**
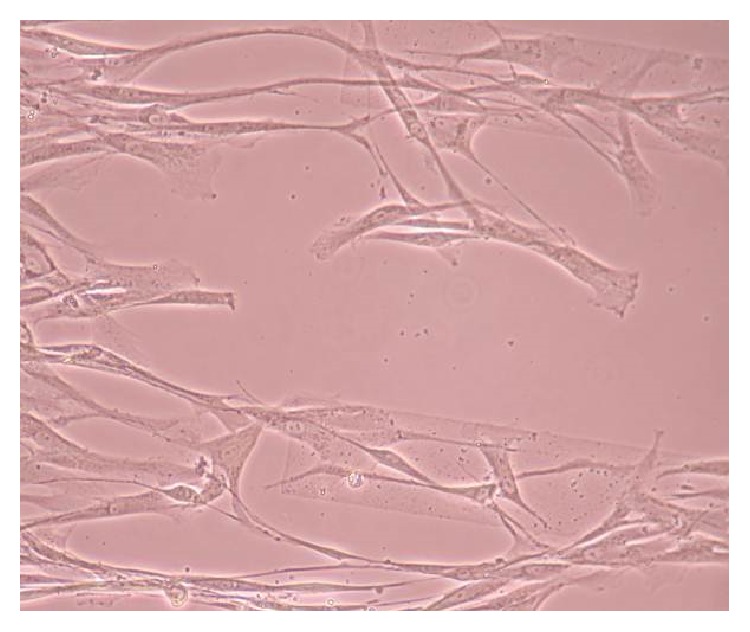
CnC on PSS micropatterns at 11 days after seeding: (PSS/PEI)_4_/PSS—100 *μ*m.

**Figure 14:**
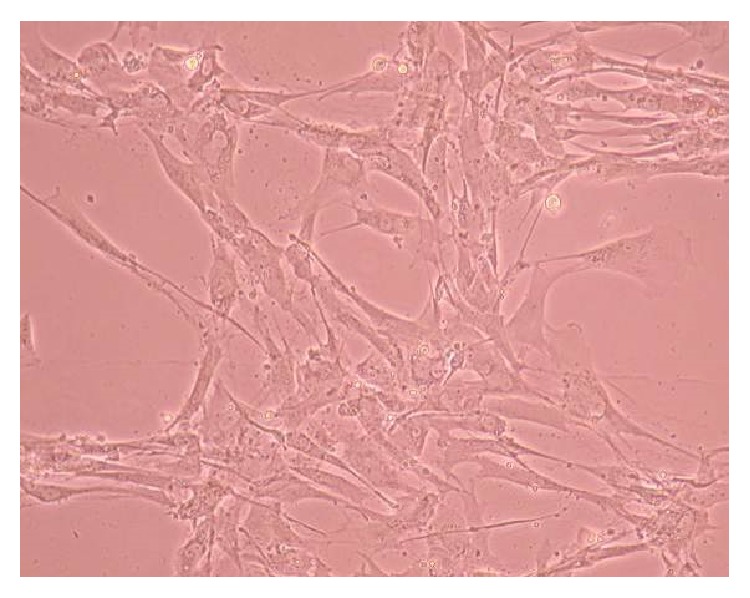
CnC on collagen micropatterns at 10 days after seeding: (PSS/Collagen)_5_—100 *μ*m.

**Figure 15:**
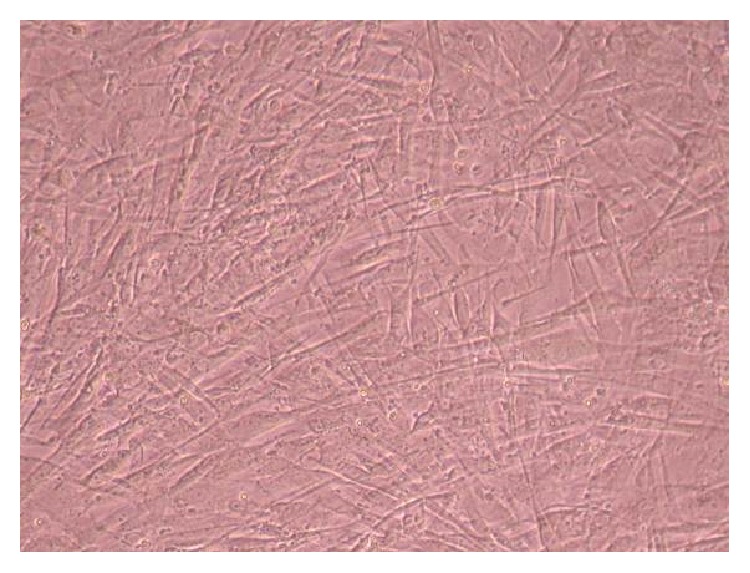
CnC on collagen micropatterns at 11 days after seeding: (PSS/Collagen)_5_.

**Figure 16:**
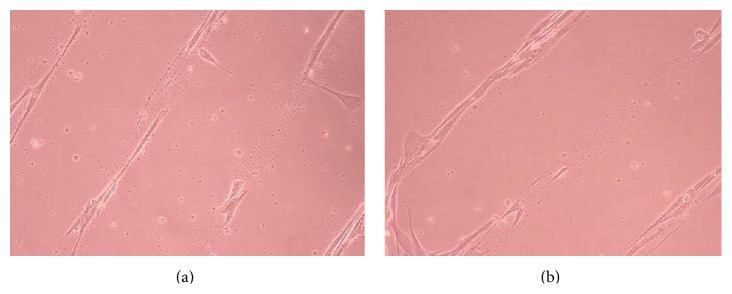
CnC on CS micropatterns at 9 days after seeding: (a) (CS/PEI)_4_/CS—80 *μ*m, (b) (CS/PEI)_9_/CS—80 *μ*m.

**Figure 17:**
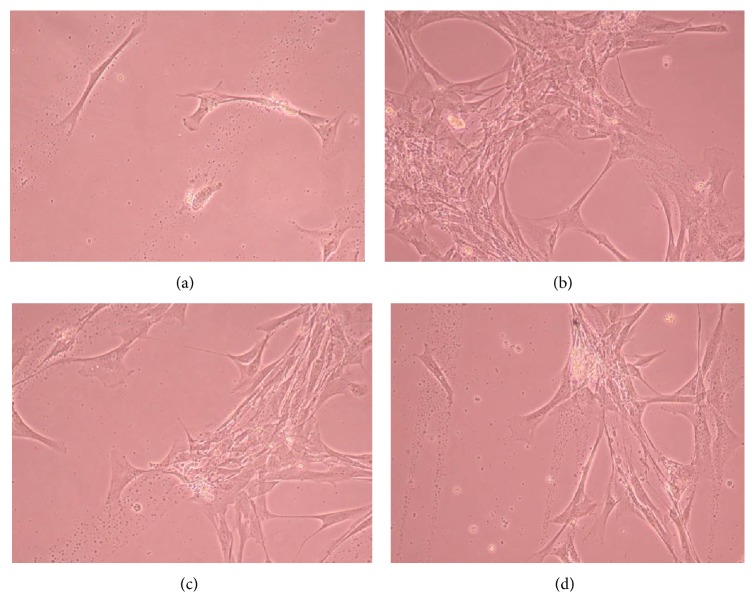
CnC on CS micropatterns at 10 days after seeding: (a) (CS/PEI)_4_/CS—80 *μ*m, (b) (CS/PEI)_4_/CS—100 *μ*m, (c) (CS/PEI)_9_/CS—100 *μ*m, and (d) (CS/PEI)_9_/CS—80 *μ*m.

**Figure 18:**
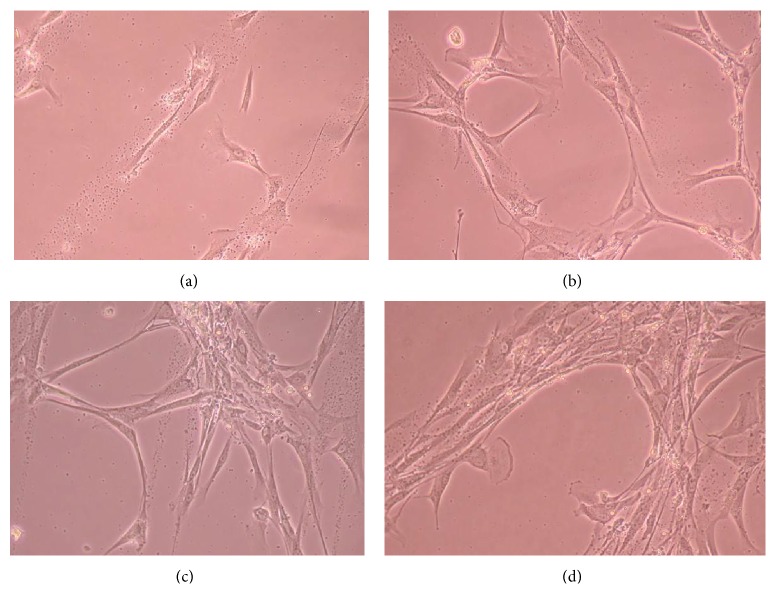
CnC on CS micropatterns at 11 days after seeding: (a) (CS/PEI)_4_/CS—80 *μ*m, (b) (CS/PEI)_4_/CS—100 *μ*m, (c) (CS/PEI)_9_/CS—80 *μ*m, and (d) (CS/PEI)_9_/CS—100 *μ*m.

Figures 11–13 demonstrate that CnC growth is mainly restricted to the PSS micropatterns. The CnC on the micropatterns were aligned along the length of the stripe patterns. From Figures 14 and 15, CnC have also grown to confluence. The confluent CnC make the collagen micropatterns very hard to be discerned in the images. From Figures 16–18, it can be observed that CnC are preferentially growing on the CS micropatterns. Also, it can be observed that CnC growing on the micropatterns are aligned along the length of the stripe patterns.

## 4. Discussion

Several similar studies have been conducted in other eukaryotic cells [11, 14, 24, 35, 40–45], and a few studies have also focused on different types of chondrocytes [46]. Also, diverse methods have been adapted to create the micropatterns required for the studies. To our knowledge, ours is the first study using canine chondrocytes as a model for directed growth on micropatterned substrates. The majority of the above-mentioned studies have reported constrained and preferential cell growth on micropatterned substrates. Specifically, protein micropatterns (bone morphogenetic protein 2 (BMP-2) printed on polystyrene (PS)) fabricated by microcontact printing significantly influenced the adhesion, spread, alignment, and functions of human chondrocytes. As in our studies and several similar studies, human chondrocytes showed preferential adhesion on the BMP-2 micropatterns. Both the shapes and sizes of the micropatterns were instrumental in influencing cell adhesion, cell morphology, the degree of spreading of the cells, and more significantly type II and VI collagen expression, thus emphasizing the importance of protein micropatterns in influencing the growth and functionality of human chondrocytes [47].

While our studies were not focused on expression of different collagen and proteoglycan types, future studies will be directed towards analysis of gene expression and protein synthesis including the level of phenotypic protein marker expression as well as the potential for long-term chondrocyte functionality on micropatterned substrates. These studies would be beneficial in understanding the influence of micropatterns generated from proteins and other biomaterials on the growth and behavior of canine chondrocytes. Incorporation of growth factors and other bioactive factors that may modulate the behavior of canine chondroctyes should also be addressed. Micropatterns generated using the LbL-LO technique used in the current study can be helpful in creating *in vitro *drug-delivery models for studying the effects of different drugs on chondrocytes of varying types (growth versus articular cartilage). Our previous work, current study, and the suggested future studies (short-term and long-term) would also be extremely useful in cartilage tissue engineering and also for creating disease-study models, studying chondrocyte involvement in degenerative changes in articular cartilage, for example.

## 5. Conclusions

Defined and restrained growth of canine chondrocytes was achieved on 5 and 10 bilayer micropatterns fabricated using the LbL-LO technique. From the morphological observations, the 5- and 10-bilayer nanofilms do not produce any apparent differences in the growth pattern of CnC. 

CnC appeared to remain confluent for a longer period of time on the thinner monolayer PDDA surface between the micropatterns compared to CnC grown on the thicker PDDA micropatterns. This suggests that 5 and 10 bilayers of micropatterned PDDA might act as cell-resistant surfaces; further studies are needed to understand this observation. CnC exhibited preferential attachment on micropatterns of PEI, PSS, collagen, and CS multilayer nanofilms. CnC growth was stable for an extended period of time on micropatterned PEI, PSS, collagen, and CS suggesting their possible use in biomedical applications.
